# Investigating the use of ionization chamber and solid‐state detectors to evaluate kerma‐area product meter accuracy under TG‐125 geometry across variable field of views

**DOI:** 10.1002/acm2.70281

**Published:** 2025-10-09

**Authors:** Atsushi Fukuda, Pei‐Jan Paul Lin

**Affiliations:** ^1^ Department of Radiological Sciences School of Health Sciences Fukushima Medical University Fukushima Japan; ^2^ Department of Radiology Virginia Commonwealth University Health System Richmond Virginia USA

**Keywords:** fluoroscopic system, kerma‐area product meter, solid‐state detector, X‐ray field dependence

## Abstract

**Background:**

A kerma‐area product (KAP) meter has been installed in a fluoroscopic system, and its accuracy has been evaluated in task group (TG)‐190 geometry using an external detector. However, it is unclear that external detectors could evaluate the KAP meter accuracy under the TG‐125 geometry.

**Purpose:**

This study investigated whether the reference air kerma rate (${\dot{K}}_{a,r}$) values of the KAP meter increase with increasing field of view (FOV) and whether an external ionization chamber and solid‐state detector (SSD) could be used to evaluate KAP meter accuracy in TG‐125 geometry.

**Methods:**

An ionization chamber and three different SSDs (Radcal AGMS‐DM+, Raysafe X2 R/F sensor, and RTI Dose Probe) were placed at the patient entrance reference point in a C‐arm fluoroscopic system, and measurements were taken at FOV settings of 18, 25, 34, and 42 cm using both the TG‐190 and TG‐125 geometries. The ${\dot{K}}_{a,r}$, incident air kerma rate (${\dot{K}}_{a,i})$, and entrance surface air kerma rate (${\dot{K}}_{a,e})$ values were recorded simultaneously and compared. The measurement data were used to calculate the backscatter factor.

**Results:**

The ${\dot{K}}_{a,r}$ increased linearly with the relative X‐ray output, and their slopes significantly increased with the FOV. The ${\dot{K}}_{a,e}$ values of the ionization chamber increased with the FOV; however, the ${\dot{K}}_{a,i}$ values of the ionization chamber, ${\dot{K}}_{a,i}$ and ${\dot{K}}_{a,e}$ values of the SSDs were not dependent on the FOV and were nearly identical to each other. Although the backscatter factor for the KAP meter and ionization chamber increased with the FOV, the backscatter for the SSDs were close to unity in all FOVs and no significant difference was observed.

**Conclusions:**

The results indicated that the ${\dot{K}}_{a,r}$ values of KAP meter were increased with the FOV. Furthermore, the SSDs could be utilized to evaluate the KAP meter accuracy in TG‐125 geometry.

## 1 INTRODUCTION

Fluoroscopic systems equipped with a physical or logical kerma‐area product (KAP) meter that displays the reference air kerma ($K_{a,r}$) and reference air kerma rate (${\dot{K}}_{a,r}$) at the patient entrance reference point (PERP), as well as the KAP ($P_{KA}$) and KAP rate (${\dot{P}}_{KA}$).^1^ $K_{a,r}$ and $P_{KA}$ are frequently employed to compare radiation doses during percutaneous coronary intervention,^2^ neurointerventional,^3^ and interventional radiology procedures.^4^ The International Electrotechnical Commission (IEC) requires KAP meters to have an accuracy within ±35%.^5^

The American Association of Physicists in Medicine (AAPM) Task Group 190 (TG‐190) developed a calibration methodology for interventional X‐ray equipment (IXE) systems that involves comparing $K_{a,r}$ or $P_{KA}$ values measured by a KAP meter and the incident air kerma ($K_{a,i}$) using external detectors.^6^ With a conventional C‐arm fluoroscopic system, the external detector is placed at PERP, which lies 15 cm from the isocenter toward the focal spot along the central X‐ray beam axis.^7^ TG‐190 recommended using copper plates as the attenuation material to reduce scattered radiation at the PERP and attaching them to a flat panel detector (FPD) to attain the tube voltage in the range of 90–100 kV, which is a free‐in‐air measurement (i.e., TG‐190 geometry). TG‐190 also recommended using a one‐point calibration with an X‐ray field of 10 cm × 10 cm at the PERP.^6^ However, because a KAP meter simultaneously detects scattered radiation from its components and the X‐ray tube assembly, the measurement accuracy of ${\dot{K}}_{a,r}$ is unclear under clinical situations. The accuracy may depend on the automatic brightness control/automatic dose rate control (ADRC) logic and the field of view (FOV). Meanwhile, AAPM Task Group 125 (TG‐125) focused on the ADRC logic in modern fluoroscopic systems.^8^ TG‐125 recommended placing the ionization chamber at the PERP and using polymethyl methacrylate (PMMA) plates as the attenuation material to evaluate the ADRC and the radiation dose to the patient (i.e., TG‐125 geometry). However, the ionization chamber measures the entrance surface air kerma rate (${\dot{K}}_{a,e}$), which comprises both ${\dot{K}}_{a,i}$ and scattered radiation from the PMMA plates. Thus, TG‐125 geometry appears not suitable for comparison or evaluation of KAP meters because $K_{a,r}$, $P_{KA}$, and $K_{a,i}$ are measured free‐in‐air.

Solid‐state detectors (SSDs), which comprise multilayer photodiodes, carrier, and filtering materials, have been employed to measure $K_{a,i}$, ${\dot{K}}_{a,i}$, and half‐value layers.^9^, ^10^ Thus, they can be utilized as external detectors to verify the ${\dot{K}}_{a,r}$ values of KAP meters in TG‐190 geometry. In addition, SSDs are manufactured with a tin, tungsten, or lead backing to reduce influence of backscattered radiation.^11^ Therefore, we hypothesized that the SSDs may also be employed to verify the ${\dot{K}}_{a,r}$ values of KAP meters under TG‐125 geometry. If our hypothesis is proved to be correct, the KAP meter accuracy could be evaluated under ADRC using various FOVs. In addition, because the TG‐272 recommended that the annual assessment of ADRC, the KAP meter and ADRC could be simultaneously evaluated under the same geometry.^12^ It will improve the workflow for medical physicists. In this study, the ${\dot{K}}_{a,r}$, ${\dot{K}}_{a,i}$, and ${\dot{K}}_{a,e}$ values were measured in TG‐190 and TG‐125 geometries across variable FOVs. Subsequently, we investigated whether the ${\dot{K}}_{a,r}$ values of the KAP meter increase with increasing FOV, and whether an external ionization chamber and SSDs could be utilized to simultaneously evaluate KAP meter accuracy and ADRC in TG‐125 geometry.

## 2 METHODS

### 2.1 Devices

A C‐arm fluoroscopic system (Ultimax‐i, Canon Medical Systems, Otawara, Japan) equipped with a FPD was employed with focal spot–PERP and maximum focal spot–FPD distances of 56 and 123 cm, respectively. The FPD had maximum FOV of 42 cm × 42 cm. The X‐ray tube could be positioned above or below the patient, and the tube voltage range was 50–110 kV while the tube current range was 10–100 mA. The fluoroscopic system had frame rates of 1, 2.14, 3.75, 7.5, and 15 frames per second (f/s) in pulsed fluoroscopy. Inherent filtration was achieved by a built‐in 1.1‐mm‐thick aluminum. The spectral shaping filter (SSF) comprised a 1.2‐mm‐thick aluminum (Al) filter, a 0.03‐mm‐thick tantalum (Ta) filter, and a 0.2‐mm‐thick copper (Cu) filter. The system could be set to FOVs of 18 cm × 18 cm, 25 cm × 25 cm, 34 cm × 34 cm, and 42 cm × 42 cm.

The chamber‐in‐chamber KAP meter (DIAMENTOR RS‐KDK, PTW Freiburg, Germany) was installed within the fluoroscopic system and could measure ${\dot{K}}_{a,r}$ with automatic temperature and pressure correction during fluoroscopic X‐ray procedures. The housing had dimensions of 183.2 mm × 163.2 mm × 17.4 mm. The specifications showed an energy dependence within ±8% for the tube voltages at 40–150 kV. To clarify the different geometries in this study, the ${\dot{K}}_{a,r:TG190}^{RS-KDK}$ and ${\dot{K}}_{a,r:TG125}^{RS-KDK}$ denote ${\dot{K}}_{a,r}$ measured with DIAMENTOR RS‐KDK under TG‐190 and TG‐125 geometries, respectively.

Table 1 shows the specifications of the external detectors employed in this study. They are: (a) a 6‐cc ionization chamber (10×6‐6, Radcal, Monrovia, CA, USA) and three SSDs: (b) the AGMS‐DM+ (Radcal, Monrovia, CA, USA), (c) X2 R/F sensor (Unfors RaySafe AB, Hovås, Sweden), and (d) RTI Dose Probe (R100B, RTI group, Mölndal, Sweden). The external detectors measured ${\dot{K}}_{a,i}$ values under TG‐190 geometry and ${\dot{K}}_{a,e}$ values under TG‐125 geometry. The ${\dot{K}}_{a,i}$ values measured by the ionization chamber and three SSDs are collectively denoted as ${\dot{K}}_{a,i}^{ext}$ and individually denoted as ${\dot{K}}_{a,i}^{10X6-6}$, ${\dot{K}}_{a,i}^{AGMS-DM+}$, ${\dot{K}}_{a,i}^{X2\;R/F\;sensor}$, and ${\dot{K}}_{a,i}^{RTI\;dose\;probe}$, respectively. The ${\dot{K}}_{a,e}$ values are collectively denoted as ${\dot{K}}_{a,e}^{ext}$ and individually denoted as ${\dot{K}}_{a,e}^{10X6-6}$, ${\dot{K}}_{a,e}^{AGMS-DM+}$, ${\dot{K}}_{a,e}^{X2\;R/F\;sensor}$, and ${\dot{K}}_{a,e}^{RTI\;dose\;probe}$, respectively.

**TABLE 1:** External detectors employed in this study.

External detectors	Detector type	Manufacturer	Size	Reference point	Measuring assembly	Software	Uncertainty of dose measurement
10×6‐6	Ion chamber	Radcal	Sensitivity volume: 6‐cc 38 mm × 25 mm*Φ* Overall size: 118 mm × 25 mm*Φ*	12.5 mm from lateral surface 13 mm from top surface	Accu‐Gold+	Accu‐Gold2	±5%
AGMS‐DM+	SSD	Radcal	35.6 mm × 20.0 mm × 11.8 mm	3.2 mm from top surface	Accu‐Gold+	Accu‐Gold2	±5%
X2 R/F sensor	SSD	Unfors RaySafe AB	14 mm × 22 mm × 79 mm	3.5 mm from top surface	Raysafe X2	RaySafe view	±5%
RTI dose probe	SSD	RTI	19.8 mm × 45.0 mm × 7.4 mm	3.5 mm from top surface	Black Piranha	Ocean professional	±4%

Abbreviation: SSD, solid‐state detector.

### 2.2 Data acquisition

**FIGURE 1:**
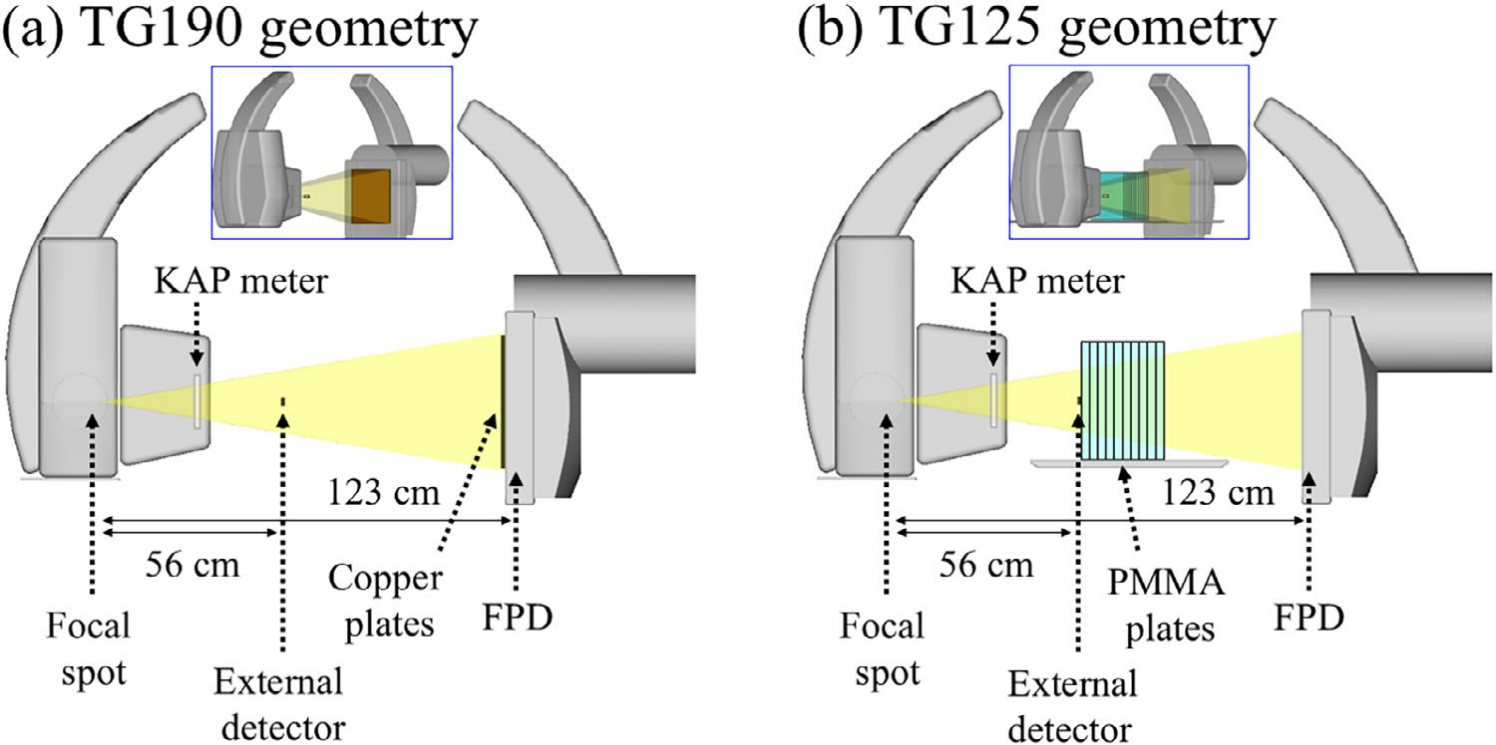
Measurement setups for the (a) TG‐190 geometry and (b) TG‐125 geometry. Insets show oblique views.

Figure 1a shows the measurement setup for the TG‐190 geometry. The X‐ray tube and FPD were laterally positioned, and the focal spot–FPD distance was set to the maximum value of 123 cm. Each external detector was placed at the PERP. ${\dot{K}}_{a,r:TG190}^{RS-KDK}$ and ${\dot{K}}_{a,i}^{ext}$ were measured at a frame rate of 15 f/s and FOVs of 18 cm × 18 cm, 25 cm × 25 cm, 34 cm × 34 cm, and 42 cm × 42 cm, using a 1.2 mm Al, 0.03 mm Ta, and 0.2 mm Cu as the SSF. Each measurement took place over 1 min. Copper plates with an area of 42 cm × 42 cm were attached to the FPD as the attenuation material and increased in thickness from 1 to 9 mm in increments of 1 mm until the maximum X‐ray output is achieved. The tube voltage, tube current, pulse width, ${\dot{K}}_{a,r:TG190}^{RS-KDK}$, and ${\dot{K}}_{a,i}^{ext}$ were recorded simultaneously.

Figure 1b shows the measurement setup for the TG‐125 geometry. The X‐ray tube, FPD, and external detectors were set to the same positions as in the TG‐190 geometry. However, the attenuation material was replaced with PMMA plates with an area of 35.5 cm × 35.5 cm and thickness of 1 in (25.4 mm). PMMA plates were added to increase the thickness in increments of 1 in just behind the external detector until the maximum X‐ray output is attained. Measurements were performed using the same X‐ray parameters as for the TG‐190 geometry. The tube voltage, tube current, pulse width, ${\dot{K}}_{a,r:TG125}^{RS-KDK}$, and ${\dot{K}}_{a,e}^{ext}$ were recorded simultaneously.

### 2.3 Data analysis

The relative X‐ray output was calculated as $V^{2}IP$ from the recorded tube voltage (*V*), tube current (*I*), and pulse width (*P*). Subsequently, ${\dot{K}}_{a,r:TG190}^{RS-KDK}$, ${\dot{K}}_{a,r:TG125}^{RS-KDK}$, ${\dot{K}}_{a,i}^{ext}$, and ${\dot{K}}_{a,e}^{ext}$ were plotted as functions of the relative X‐ray output. The slopes and standard errors of these functions in the TG‐190 and TG‐125 geometries were calculated and compared by an analysis of covariance (ANCOVA). These slopes were also employed to determine the backscatter factor (BSF) of the KAP meter ($BSF^{RS-KDK}$) and external detectors ($BSF^{ext}$), as shown in the following equations.

(1) $$\begin{array}{cc} BSF^{RS-KDK} & =\frac{{\dot{K}}_{a,r:TG125}^{RS-KDK}}{{\dot{K}}_{a,r:TG190}^{RS-KDK}}=\frac{S_{TG125}^{RS-KDK}}{S_{TG190}^{RS-KDK}}\\ BSF^{ext} & =\frac{{\dot{K}}_{a,e}^{ext}}{{\dot{K}}_{a,i}^{ext}}=\frac{S_{TG125}^{ext}}{S_{TG190}^{ext}} \end{array}$$

where $S_{TG125}^{RS-KDK}$, $S_{TG190}^{RS-KDK}$, $S_{TG125}^{ext}$, and $S_{TG190}^{ext}$ are the slopes using the KAP meter or an external detector, respectively, for a given FOV and geometry. All statistical analyses were performed using the R software package for Windows (version 4.4.1),^13^ and a *p*‐value of < 0.05 was considered statistically significant.

## 3 RESULTS

**FIGURE 2:**
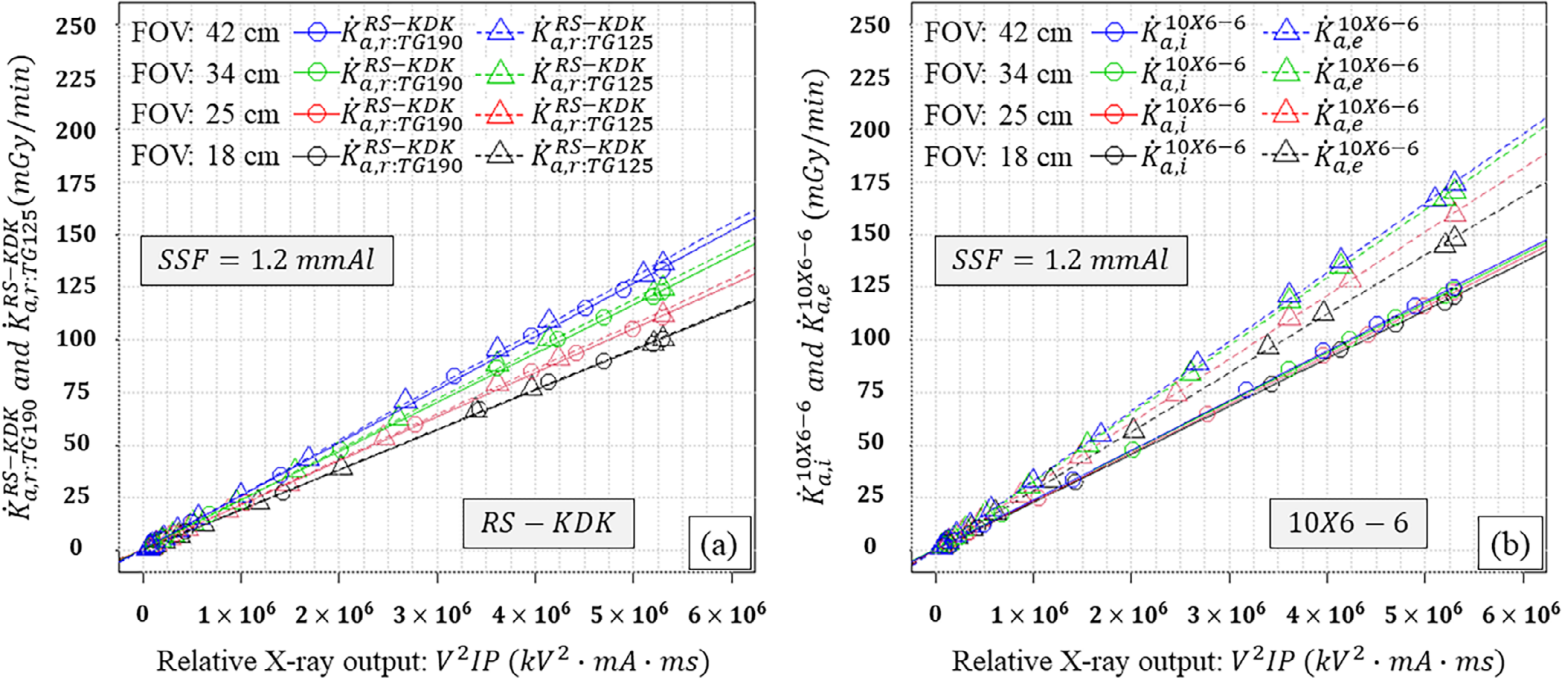
Results using the 10×6‐6 ionization chamber as the external detector: (a) ${\dot{K}}_{a,r:TG190}^{RS-KDK}$ and ${\dot{K}}_{a,r:TG125}^{RS-KDK}$, and (b) ${\dot{K}}_{a,i}^{10X6-6}$ and ${\dot{K}}_{a,e}^{10X6-6}$ as functions of the relative X‐ray output and field of view (FOV) when a 1.2 mm Al spectral shaping filter (SSF) was employed.

Figure 2 shows the results for the KAP meter (${\dot{K}}_{a,r:TG190}^{RS-KDK}$, ${\dot{K}}_{a,r:TG125}^{RS-KDK}$) and ionization chamber as the external detector (${\dot{K}}_{a,i}^{10X6-6}$, ${\dot{K}}_{a,e}^{10X6-6}$) as functions of the relative X‐ray output and FOV when a 1.2 mm Al SSF was employed. For the KAP meter, both ${\dot{K}}_{a,r:TG190}^{RS-KDK}$ and ${\dot{K}}_{a,r:TG125}^{RS-KDK}$ increased linearly with the relative X‐ray output. Their slopes increased with the FOV, and the slopes of the ${\dot{K}}_{a,r:TG190}^{RS-KDK}$ and ${\dot{K}}_{a,r:TG125}^{RS-KDK}$ differed significantly at FOVs of 25, 34, and 42 cm (*p* < 0.05). For the ionization chamber, ${\dot{K}}_{a,i}^{10X6-6}$ increased linearly with the relative X‐ray output but remained consistent across different FOVs. Because the copper plates were attached to the FPD under TG‐190 geometry, the scattered radiation had no influence on ${\dot{K}}_{a,i}^{10X6-6}$. Conversely, ${\dot{K}}_{a,e}^{10X6-6}$ was obviously larger than ${\dot{K}}_{a,i}^{10X6-6}$ for a given relative X‐ray output and increased with FOV. The ionization chamber was attached to the PMMA plates under TG‐125 geometry. Therefore, the backscatter had influence on the ${\dot{K}}_{a,e}^{10X6-6}$. The slopes of ${\dot{K}}_{a,i}^{10X6-6}$ and ${\dot{K}}_{a,e}^{10X6-6}$ differed significantly at all FOVs (*p* < 0.001)

**FIGURE 3:**
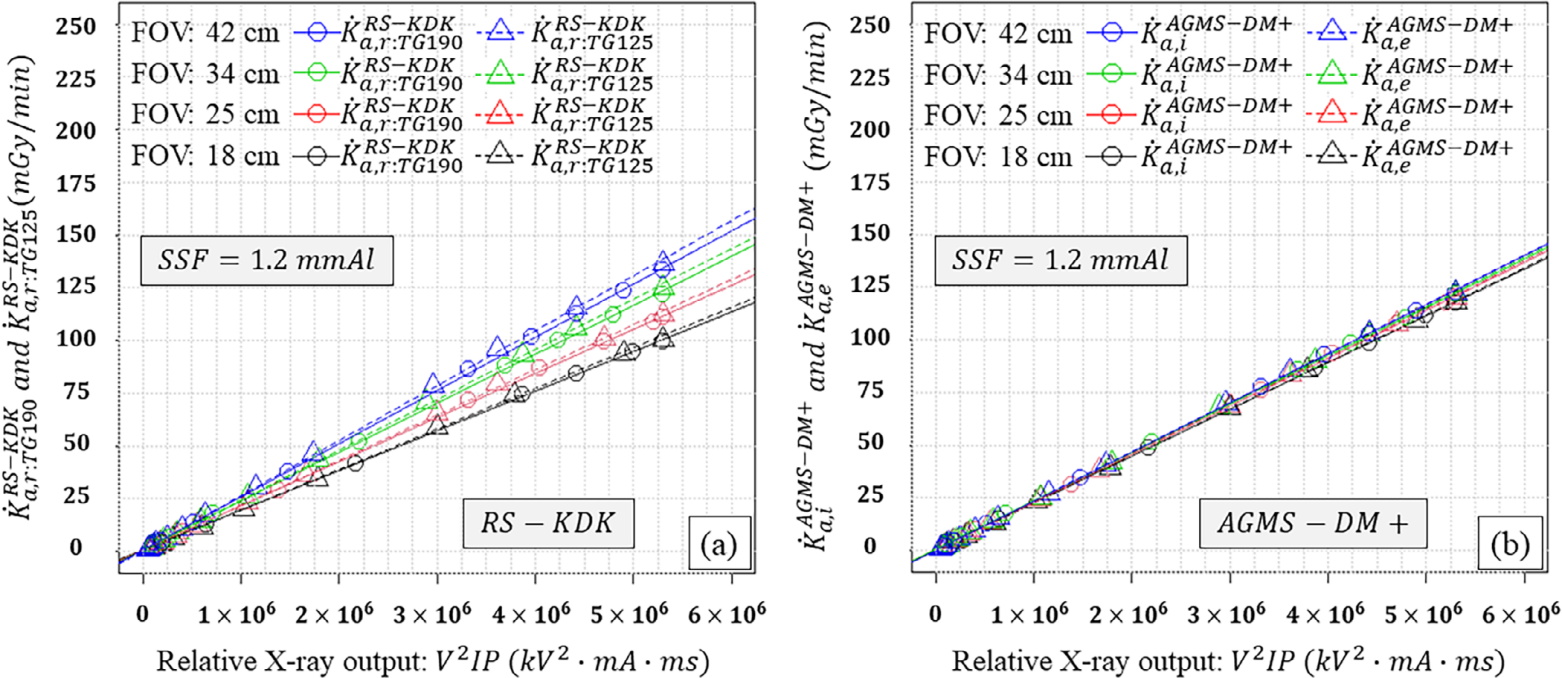
Results using AGMS‐DM+ as the external detector: (a) ${\dot{K}}_{a,r:TG190}^{RS-KDK}$ and ${\dot{K}}_{a,r:TG125}^{RS-KDK}$, and (b) ${\dot{K}}_{a,i}^{AGMS-DM+}$ and ${\dot{K}}_{a,e}^{AGMS-DM+}$ as functions of the relative X‐ray output and field of view (FOV) when a 1.2 mm Al spectral shaping filter (SSF) was employed.

**FIGURE 4:**
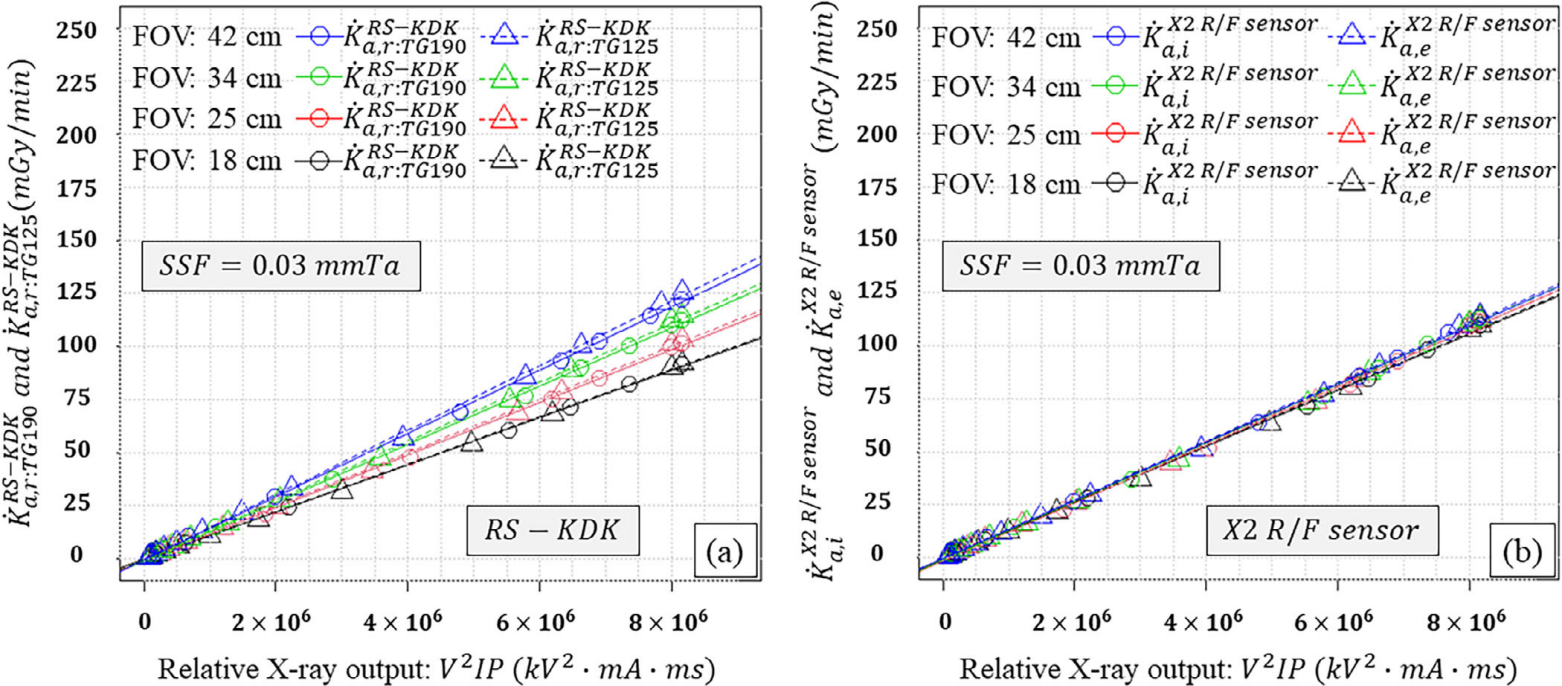
Results using X2 R/F sensor as the external detector: (a) ${\dot{K}}_{a,r:TG190}^{RS-KDK}$ and ${\dot{K}}_{a,r:TG125}^{RS-KDK}$, and (b) ${\dot{K}}_{a,i}^{X2\;R/F\;sensor}$ and ${\dot{K}}_{a,e}^{X2\;R/F\;sensor}$ as functions of the relative X‐ray output and field of view (FOV) when a 0.03 mm Ta spectral shaping filter (SSF) was employed.

**FIGURE 5:**
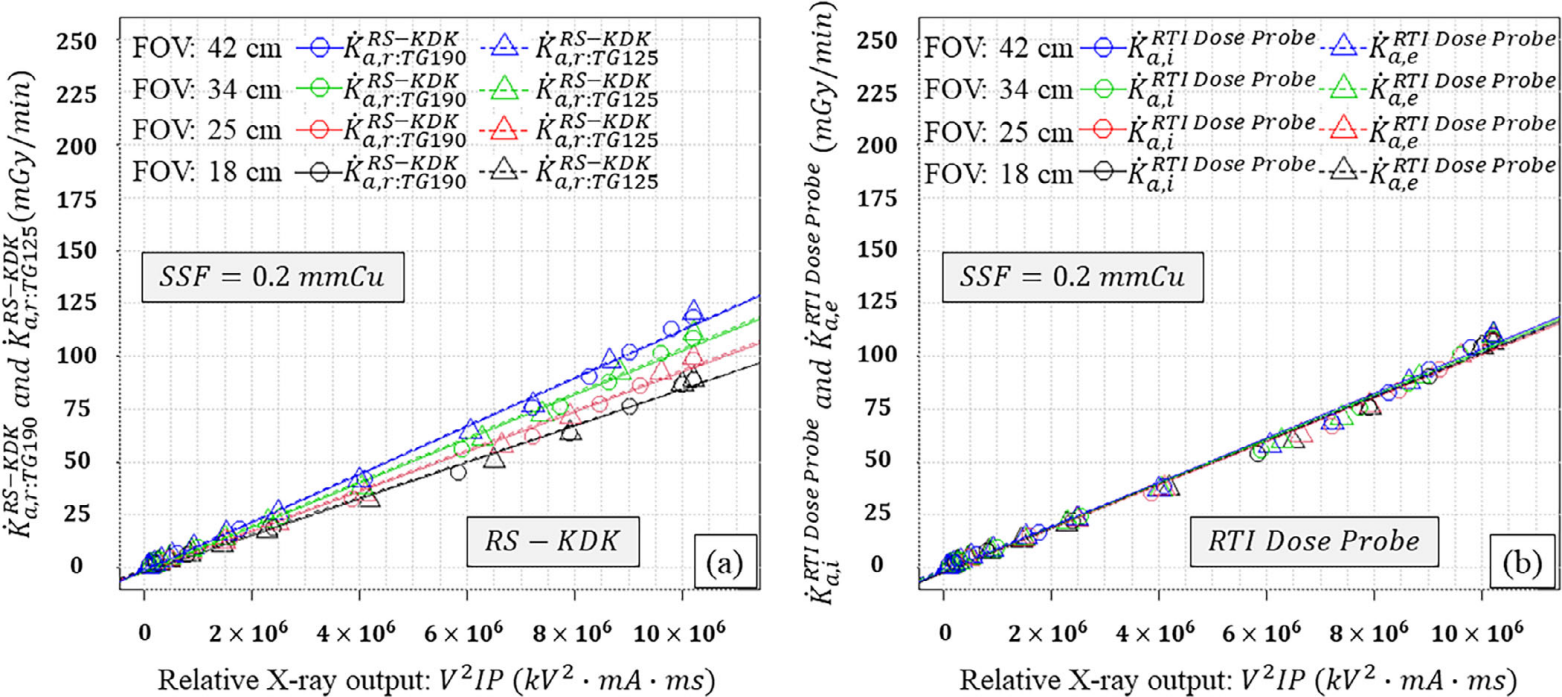
Results using RTI Dose Probe as the external detector: (a) ${\dot{K}}_{a,r:TG190}^{RS-KDK}$ and ${\dot{K}}_{a,r:TG125}^{RS-KDK}$, and (b) ${\dot{K}}_{a,i}^{RTI\;Dose\;Probe}$ and ${\dot{K}}_{a,e}^{RTI\;Dose\;Probe}$ as functions of the relative X‐ray output and field of view (FOV) when a 0.2 mm Cu spectral shaping filter (SSF) was employed.

Figures 3, 4, 5 similarly show the results for the KAP meter (${\dot{K}}_{a,r:TG190}^{RS-KDK}$, ${\dot{K}}_{a,r:TG125}^{RS-KDK}$) with different SSDs as the external detector (${\dot{K}}_{a,i}^{AGMS-DM+}$, ${\dot{K}}_{a,e}^{AGMS-DM+}$, ${\dot{K}}_{a,i}^{X2\;R/F\;sensor}$, ${\dot{K}}_{a,e}^{X2\;R/F\;sensor}$, ${\dot{K}}_{a,i}^{RTI\;dose\;probe}$, and ${\dot{K}}_{a,e}^{RTI\;dose\;probe}$) as functions of the relative X‐ray output, FOV, and three SSFs. In comparison with the results as shown in Figure 2a, ${\dot{K}}_{a,r:TG190}^{RS-KDK}$ and ${\dot{K}}_{a,r:TG125}^{RS-KDK}$ were reduced as a function of the SSFs. For the SSDs, ${\dot{K}}_{a,i}^{AGMS-DM+}$, ${\dot{K}}_{a,e}^{AGMS-DM+}$, ${\dot{K}}_{a,i}^{X2\;R/F\;sensor}$, ${\dot{K}}_{a,e}^{X2\;R/F\;sensor}$, ${\dot{K}}_{a,i}^{RTI\;dose\;probe}$, and ${\dot{K}}_{a,e}^{RTI\;dose\;probe}$ linearly increased with the relative X‐ray output and were almost identical to each other.

Table 2 lists the BSFs calculated with Equation (1). The $BSF^{RS-KDK}$ increased with the FOV when 1.2 mm Al SSF was employed. The $BSF^{10X6-6}$ increased with the FOV regardless of SSFs. However, interestingly, the $BSF^{AGMS-DM+}$, $BSF^{X2\;R/F\;sensor}$, and $BSF^{RTI\;Dose\;Probe}$ were almost unity in all FOVs and did not differ significantly. The standard errors in 0.2 mm Cu SSF were larger than those in 1.2 mm Al and 0.03 mm Ta SSFs because the relationship between ${\dot{K}}_{a,i}^{ext}$ or ${\dot{K}}_{a,e}^{ext}$ and $V^{2}IP$ was somewhat non‐linear in 0.2 mm Cu SSF as shown in Figure 5.

**TABLE 2:** Backscatter factors measured by the KAP meter and external detectors.

		DIAMENTOR RS‐KDK versus 10×6‐6		DIAMENTOR RS‐KDK versus AGMS DM+		DIAMENTOR RS‐KDK versus X2 R/F sensor		DIAMENTOR RS‐KDK versus RTI dose probe	
SSF	FOV (cm)	$BSF^{RS-KDK}$	$BSF^{10X6-6}$	$BSF^{RS-KDK}$	$BSF^{AGMS\;DM+}$	$BSF^{RS-KDK}$	$BSF^{X2\;R/F\;sensor}$	$BSF^{RS-KDK}$	$BSF^{RTI\;Dose\;Probe}$
1.2 mm Aluminum	18	1.01 ± 0.01	1.23 ± 0.01^*^	1.02 ± 0.01	1.01 ± 0.00	1.03 ± 0.01^*^	1.01 ± 0.01	1.02 ± 0.01^*^	1.01 ± 0.01
	25	1.02 ± 0.01^*^	1.31 ± 0.00^*^	1.02 ± 0.01^*^	1.00 ± 0.01	1.04 ± 0.01^*^	1.01 ± 0.01	1.03 ± 0.01^*^	1.01 ± 0.01
	34	1.02 ± 0.01^*^	1.38 ± 0.01^*^	1.03 ± 0.01^*^	1.00 ± 0.00	1.03 ± 0.01^*^	1.01 ± 0.01	1.03 ± 0.01^*^	1.01 ± 0.01
	42	1.03 ± 0.01^*^	1.40 ± 0.01^*^	1.03 ± 0.01^*^	1.00 ± 0.01	1.03 ± 0.01^*^	1.00 ± 0.01	1.03 ± 0.01^*^	1.00 ± 0.01
0.03 mm Tantalum	18	1.02 ± 0.01	1.28 ± 0.02^*^	1.00 ± 0.01	0.99 ± 0.01	1.01 ± 0.01	1.00 ± 0.02	0.99 ± 0.01	0.99 ± 0.01
	25	1.02 ± 0.01	1.35 ± 0.02^*^	1.01 ± 0.01	1.00 ± 0.01	1.01 ± 0.01	1.00 ± 0.02	1.00 ± 0.01	0.99 ± 0.01
	34	1.01 ± 0.01	1.39 ± 0.02^*^	1.02 ± 0.01	1.00 ± 0.01	1.02 ± 0.01	1.00 ± 0.01	1.01 ± 0.01	1.00 ± 0.01
	42	1.03 ± 0.01^*^	1.45 ± 0.02^*^	1.02 ± 0.01	1.00 ± 0.01	1.03 ± 0.01^*^	1.00 ± 0.01	1.02 ± 0.01^*^	1.01 ± 0.01
0.2 mm copper	18	1.00 ± 0.03	1.19 ± 0.05^*^	1.00 ± 0.04	0.99 ± 0.04	0.99 ± 0.03	0.98 ± 0.04	0.99 ± 0.03	0.98 ± 0.04
	25	0.99 ± 0.03	1.25 ± 0.05^*^	1.00 ± 0.03	1.00 ± 0.04	0.99 ± 0.03	0.98 ± 0.04	1.01 ± 0.03	1.01 ± 0.03
	34	1.00 ± 0.03	1.33 ± 0.04^*^	1.00 ± 0.03	0.98 ± 0.03	1.00 ± 0.03	0.98 ± 0.03	1.00 ± 0.03	0.99 ± 0.03
	42	1.02 ± 0.03	1.40 ± 0.04^*^	1.00 ± 0.03	0.97 ± 0.03	1.01 ± 0.03	0.99 ± 0.03	1.00 ± 0.03	0.98 ± 0.03

Abbreviations: BSF, backscatter factor; FOV, field of view; KAP, kerma area product; SSF, spectral shaping filter.

Means and standard errors are calculated.

BSFs were calculated as $BSF^{RS-KDK}=\frac{{\dot{K}}_{a,r:TG125}^{RS-KDK}}{{\dot{K}}_{a,r:TG190}^{RS-KDK}}=\frac{S_{TG125}^{RS-KDK}}{S_{TG190}^{RS-KDK}}$ and $BSF^{ext}=\frac{{\dot{K}}_{a,e}^{ext}}{{\dot{K}}_{a,i}^{ext}}=\frac{S_{TG125}^{ext}}{S_{TG190}^{ext}}$, respectively.

* Statistical difference between $S_{TG125}^{RS-KDK}$ and $S_{TG190}^{RS-KDK}$, or $S_{TG125}^{ext}$ and $S_{TG190}^{ext}$ (*p* < 0.05).

## 4 DISCUSSION

This study investigated whether the ${\dot{K}}_{a,r}$ values of the KAP meter increase with increasing FOV, and whether an external ionization chamber and SSDs could be utilized to evaluate KAP meter accuracy in TG‐125 geometry. Although ${\dot{K}}_{a,r:TG190}^{RS-KDK}$, ${\dot{K}}_{a,r:TG125}^{RS-KDK}$, and ${\dot{K}}_{a,e}^{10X6-6}$ increased with the FOV, ${\dot{K}}_{a,i}^{10X6-6}$, ${\dot{K}}_{a,i}^{AGMS-DM+}$, ${\dot{K}}_{a,e}^{AGMS-DM+}$, ${\dot{K}}_{a,i}^{X2\;R/F\;sensor}$, ${\dot{K}}_{a,e}^{X2\;R/F\;sensor}$, ${\dot{K}}_{a,i}^{RTI\;dose\;probe}$, and ${\dot{K}}_{a,e}^{RTI\;dose\;probe}$ were all independent of the FOV and almost identical to each other. These results indicate that the ionization chamber could be utilized to verify the KAP meter in the TG‐190 geometry, while the SSDs could be utilized to verify the KAP meter in both TG‐190 and TG‐125 geometries.

The slopes of ${\dot{K}}_{a,r:TG190}^{RS-KDK}$ and ${\dot{K}}_{a,r:TG125}^{RS-KDK}$ were increased with the FOV (Figures 2, 3, 4, 5). This is because increasing the FOV, increased the off‐focus radiation led to an increase in scattered radiation into the KAP meter.^14^ Furthermore, the slopes of ${\dot{K}}_{a,r:TG190}^{RS-KDK}$ and ${\dot{K}}_{a,r:TG125}^{RS-KDK}$ differed for a given FOV, which can be attributed to the scattered radiation from the PMMA plates.

In this study, an ionization chamber (10×6‐6) and three different SSDs (AGMS‐DM+, X2 R/F sensor, and RTI Dose Probe) were employed as external detectors. As predicted, ${\dot{K}}_{a,e}^{10X6-6}$ was affected by the scattered radiation from the PMMA plates. However, the slopes of ${\dot{K}}_{a,i}^{10X6-6}$, ${\dot{K}}_{a,i}^{AGMS-DM+}$, ${\dot{K}}_{a,e}^{AGMS-DM+}$, ${\dot{K}}_{a,i}^{X2\;R/F\;sensor}$, ${\dot{K}}_{a,e}^{X2\;R/F\;sensor}$, ${\dot{K}}_{a,i}^{RTI\;dose\;probe}$, and ${\dot{K}}_{a,e}^{RTI\;dose\;probe}$ did not differ significantly. These results indicate that ${\dot{K}}_{a,i}^{ext}$ was not affected by scattered radiation from the copper plates, and the SSDs were minimally or not affected by scattered radiation from both PMMA and copper plates. Thus, the SSDs can be used to verify KAP meters using the TG‐125 geometry.

As mentioned above, TG‐190 recommended using a one‐point calibration with an X‐ray field of 10 cm × 10 cm at the PERP.^6^ However, because the accuracy of the KAP meter depended on the FOV in this study, the X‐ray field dependent calibration factor might improve the accuracy of the KAP meter. Fortunately, the chamber‐in‐chamber KAP meter has two chambers to measure the $K_{a,r}$ and $P_{KA}$. Because the X‐ray field at PERP can be simply calculated as $P_{KA}/K_{a,r}$, the X‐ray field dependent calibration factor might be developed to compensate for changes in the X‐ray field with the FOV and focal spot–FPD distance.^15^ It should also be pointed out that the $P_{KA}/K_{a,r}$ displayed by the IXE systems are normally measured and calibrated under a given standard FOV specific to the IXE unit of various IXE manufacturers (typically 20–30 cm FOV) and scaled for other FOVs in accordance to each manufacturer's default setup and calibration procedures.

This study had two limitations. First, measurements were performed using a single KAP meter; however, many types of KAP meters are compatible with fluoroscopic systems, and they may result in differences in the ADRC logic and the X‐ray field dependence. Second, three SSDs were investigated in this study. Because there are many types of SSDs available commercially, the SSDs without high‐Z backing material may not be employed to measure ${\dot{K}}_{a,e}$ in TG‐125 geometry. Therefore, the $BSF^{ext}$ for the other SSDs should be measured and evaluated before employed for verification measurements of ${\dot{K}}_{a,r}$ values of KAP meters.

## 5 CONCLUSION

The results indicated that the ${\dot{K}}_{a,r}$ values of the KAP meter increased with increasing FOV, motivating measurement beyond the one FOV calibration point. The results also indicated that SSDs could be utilized to simultaneously evaluate the KAP meter accuracy and ADRC in TG‐125 geometry to adhere to TG‐272 report recommendations and improve workflow for medical physicists.

## AUTHOR CONTRIBUTIONS

**Atsushi Fukuda**: Conception and design of the study, analysis and interpretation of data, collection and assembly of data, drafting of the article, and final approval of the article. **Pei‐Jan Paul Lin**: Conception and design of the study, analysis and interpretation of data, and final approval of the article.

## CONFLICT OF INTEREST STATEMENT

The authors have no relevant conflicts of interest to disclose.

## Data Availability

The data that support the findings of this study are available from the corresponding author upon reasonable request.
